# Mortality of Cities

**Published:** 1874-03

**Authors:** 


### MORTALITY OF CITIES.

In Philadelphia one person dies out of every fifty, annually.

| | | | |
|---|---|---|---|
| Lowell | 1 in 50 | New York | 1 in 27 |
| Charleston, S. C. | 1 in 48 | Belgium (country) | 1 in 46 |
| London | 1 in 41 | " (town) | 1 in 36 |
| Baltimore | 1 in 40 | Sweden (country) | 1 in 44 |
| Boston | 1 in 39 | " (town) | 1 in 28 |
| Paris | 1 in 33 | France (country) | 1 in 40 |
| Savannah | 1 in 33 | Paris (town) | 1 in 33 |
| Berlin | 1 in 32 | In all England | 1 in 46 |
| Liverpool | 1 in 29 | Surry county, near London | 1 in 53 |
| Manchester | 1 in 29 | | |

The general practical fact apparent from these tables is, that the country is twenty-five feet per cent. more healthy than the city.

There is no large city in the civilized world, which has facilities of air, water and drainage equal to New York, yet more people die every year in New York, in proportion to the number of the inhabitants, than in any other city named on the list. We account for this extraordinary result in the fact of the crowded condition of the population, which most contributes to this alarming mortality. We need not be surprised at the result, when to this is added the fact that multitudes live in cellars, from one to three feet under ground, and these mainly in the lowest parts of the city, where the drainage finds its level, and where narrow streets are covered with moist filth from two to a dozen inches in depth--and in some places are encumbered with piles of corrupting rottenness three feet high, and undisturbed for months in succession.

That the crowded condition of the houses of the masses is more prominently the cause of our greater mortality, is deducible from the fact, that Philadelphia—not much cleaner than New York, with not half the advantages of air, and water, and natural drainage, having scarcely more than half our mortality, —has one house for every six and a half persons, while New York has one house for every thirteen and a half. Boston has nine, St. Louis and Cincinnati, each eight. That crowded apartments have death-dealing tendencies, more instantaneous sometimes than a plague, the incidents of the Black-hole of Calcutta afford a terrible warning; where, just a hundred and one years ago, a hundred and thirty-six persons were crowded into a room twenty feet square, with only one small window. In the morning, one hundred and twenty-three of these were piled up, a putrefying mass of dead men; only twenty-three gasping, distorted living corpses staggered out of the dreadful charnel house.

With the greatest earnestness then, do we impress upon every citizen, and every head of a family, to secure for themselves and for those under them, at least proper sleeping apartments, for in these we spend the larger and more exposed portion of our daily in-door life.

At any cost within our means, the rooms we sleep in should be the largest, highest, lightest, cleanliest, dryest and most barren of furniture in the whole house.

